# National Pension Fund for Hospital Officials and Nurses

**Published:** 1887-03-26

**Authors:** 


### A National Pension Fund for Hospital Officials and Nurses.

### The Views of Officials, Sisters, Nurses, and Others.

### By Mrs. E. A. Bedingfeld,

Lady Superintendent, The Belgravia Institute for Trained Nurses.

May I be permitted to say a few words regarding the proposed "National Annuity Fund for Trained Nurses"? I have read carefully the rules and regulations of the Fund already in existence, and have spoken to several nurses on the subject. They all see the great utility of such a Fund, but deprecate it being made so completely a charity as the said Fund makes it. Further, they are averse to the insistence on *three* years' Hospital or Infirmary training being requisite to make them eligible as annuitants. Some hospitals do not train their nurses for so long a time as three years, and therefore nurses certified from these, however good their work, would be ineligible! Moreover, there are two most useful classes who, by this rule, would be *entirely debarred* from receiving an annuity, viz., midwives and monthly nurses. The maximum period of training for midwives is *six months*. In the London Lying-in Hospitals it is only three months; and for monthly nurses, two months' training is considered sufficient. The life of a monthly nurse in constant work is harder in many ways than that of a general nurse, as she gets continuous broken nights' rest, with but little chance of recuperating her strength by sleep during the day. I can say from personal experience that a monthly nurse rarely gets more than three hours' consecutive sleep during the whole of the puerperal month. Midwives, again, are specially liable to illness, as they must go out in all weathers, and in country districts have to walk long distances—I know one who had to walk *five miles* to a case, in the snow of the 26th and 27th of December last year! Therefore they would in but few cases be able to work for the fifteen years necessary by the rules of the existing Fund to entitle them to become annuitants. It seems to me that some such scheme as the following might, if elaborated by a wiser head than mine, be the better thing.

I. Let any annual subscriptions and donations from persons not nurses, be made the nucleus for providing annuities for trained nurses who have attained a certain age, say fifty, and relief in illness if funds permit.

II. Let all nurses who can produce a genuine hospital certificate of efficient training in their respective branches be eligible; but should such certificate be of old date, let a recent reference or references be required.

III. Let each nurse subscribe a stated sum per annum, according to an arranged scale; age, and amount of annuity required, being taken into consideration in forming the scale.

IV. Let there be first, second, and third-class annuities, of different amounts, as all nurses could not afford to pay at the same rate.

V. Should any nurse die before her annuity becomes payable, or should she, from marriage or other cause, desire to retire from the Fund, let her, or in case of death, her next-of-kin, receive (as is done in the case of Life Insurance Policies) a "surrender value," *i.e.*, an agreed proportion of the money paid in by her, say two-thirds of her payments.

I believe that many nurses would subscribe to such a Fund as this willingly, and the £15 required by the existing Society to be paid by the nurse when elected, would, instead of being put into the Post Office Savings Bank, from which it could at any time be so easily drawn out for other purposes, be more safely lodged, by regular quarterly or monthly instalments, in the hands of the trustees of the Fund, from whence it could not be withdrawn.

The question of interest on deposits, or an occasional bonus to be added to the annuity, would be a question which would arise later, when the success of the Fund was assured. Provision should also be made, if possible, for helping nurses who, having subscribed for a certain number of years, become incapacitated before their annuity commences.

---

### By Four Hospital Nurses.

We are very much interested in the suggestion of "a scheme of a provident nature" in connection with a Trained Nurses' Annuity Fund, and have long felt that it is a great want, which, if we had friends who would take so much trouble, might be met by some system "established on purely commercial grounds," or in any way by which we could help ourselves in securing provision for the future. Will you kindly allow us to express our hope that the efforts of those who are interesting themselves on our behalf may result in a great success, and that this Jubilee year and the present time might be *the* opportunity for enabling hospital workers and sympathisers with them to show their appreciation, and give their support towards founding a National Provident Fund, which we believe would not fail to have as subscribers to it the very great majority of the body of workers to which we belong; and we cannot but think, with a "Hospital Sister," that "it would be found that many do care about it, though unable to move in the matter." We cannot offer any suggestion, and are quite ignorant how it could be made "a successful commercial undertaking"; but we have often wondered why some such scheme as a Friendly Society, to which a patient will often say he belongs, could not be adopted as a means of self-help, or mutual assistance, amongst the ten thousand nurses who are calculated to be now in the United Kingdom.

The present "Trained Nurses' Annuity Fund" does not seem to supply a want, and it has rules that must prevent many from sympathising with the promoters of it. One especially seems hard: a nurse thoroughly worn out, and at least fifty years of age, must pay £15 if she is successful in obtaining an annuity of £15, so that we might say that she would be for one year worse off than she was before. It may be right from a business point of view, but it does not enlist any of our better feelings in support of it.

We venture to address you, because we feel we owe thanks to you and to those who have asked you to allow space in The Hospital for the discussion of views on a subject which we are so anxious to see warmly taken up and brought to a successful issue.

The General Infirmary, Dewsbury.

